# Detection of early cartilage degeneration in the tibiotalar joint using 3 T gagCEST imaging: a feasibility study

**DOI:** 10.1007/s10334-020-00868-y

**Published:** 2020-07-28

**Authors:** Daniel B. Abrar, Christoph Schleich, Karl Ludger Radke, Miriam Frenken, Julia Stabinska, Alexandra Ljimani, Hans-Jörg Wittsack, Gerald Antoch, Bernd Bittersohl, Tobias Hesper, Sven Nebelung, Anja Müller-Lutz

**Affiliations:** 1 Department of Diagnostic and Interventional Radiology, Medical Faculty, University Hospital Düsseldorf, University Dusseldorf, Moorenstraße 5, 40225 Düsseldorf, Germany; 2 Department of Orthopedic and Trauma Surgery, Medical Faculty, Heinrich-Heine University Düsseldorf, Düsseldorf, Germany

**Keywords:** Cartilage, Magnetic resonance imaging, Proteoglycans, Osteoarthritis, Molecular imaging

## Abstract

**Objective:**

To establish and optimize a stable 3 Tesla (T) glycosaminoglycan chemical exchange saturation transfer (gagCEST) imaging protocol for assessing the articular cartilage of the tibiotalar joint in healthy volunteers and patients after a sustained injury to the ankle.

**Methods:**

Using Bloch–McConnell simulations, we optimized the sequence protocol for a 3 T MRI scanner for maximum gagCEST effect size within a clinically feasible time frame of less than 07:30 min. This protocol was then used to analyze the gagCEST effect of the articular cartilage of the tibiotalar joint of 17 healthy volunteers and five patients with osteochondral lesions of the talus following ankle trauma. Reproducibility was tested with the intraclass correlation coefficient.

**Results:**

The mean magnetization transfer ratio asymmetry (MTR_asym_), i.e., the gagCEST effect size, was significantly lower in patients than in healthy volunteers (0.34 ± 1.9% vs. 1.49 ± 0.11%; *p* < 0.001 [linear mixed model]). Intra- and inter-rater reproducibility was excellent with an average measure intraclass correlation coefficient (ICC) of 0.97 and a single measure ICC of 0.91 (*p* < 0.01).

**Discussion:**

In this feasibility study, pre-morphological tibiotalar joint cartilage damage was quantitatively assessable on the basis of the optimized 3 T gagCEST imaging protocol that allowed stable quantification gagCEST effect sizes across a wide range of health and disease in clinically feasible acquisition times.

**Electronic supplementary material:**

The online version of this article (10.1007/s10334-020-00868-y) contains supplementary material, which is available to authorized users.

## Introduction

To this day and age, several magnetic resonance imaging (MRI) techniques have emerged that go beyond mere morphological depiction of joint cartilage. Such compositional MRI techniques allow the detection of early degenerative changes of the articular cartilage, e.g., loss of proteoglycans, that precede morphological damage and hence are considered an early, and more importantly, reversible, stage of osteoarthritis (OA) [1]. Because of its proteoglycan-specifity, the gold-standard technique of compositional MRI of cartilage is delayed gadolinium-enhanced MRI of cartilage (dGEMRIC) [2, 3]. However, due to recent restrictions imposed on gadolinium-based contrast agents, alternative compositional MRI techniques that do not rely on the administration of contrast agents have received ever-increasing scientific and clinical attention [4]. Among these techniques, glycosaminoglycan chemical exchange saturation transfer (gagCEST) imaging assesses the specific GAG content in human articular cartilage and its depletion, which is considered an early sign of cartilage degeneration [5].

GagCEST imaging is based upon the chemical exchange of water protons between GAG and bulk water molecules. To induce a CEST effect, solute protons are saturated by a frequency-specific radiofrequency (RF) pulse and then transferred to bulk water by chemical exchange, which consequently reduces its signal. The normalized signal can then be used to quantify the CEST effect at a GAG -specific frequency range of 0.9–1.9 ppm via analysis of the magnetization transfer ratio asymmetry (MTR_asym_), which correlates with the GAG concentration [5, 6]. For additional details on the basic principles of CEST imaging, the interested reader is referred to earlier excellent reviews.[7, 8]. Several studies showed promising results using gagCEST imaging at the spine [9–12]. However, data on the joints of the lower extremity with substantially thinner cartilage are sparse. In 2016, our group demonstrated promising results for the application of gagCEST at the knee joint [13]. Kogan et al. applied gagCEST imaging on a 7 T MRI scanner to assess the ankle joint of healthy volunteers [14]. Even though these results were promising, gagCEST imaging of the ankle joint has not yet been established on a 3 T MRI scanner. To achieve a more widespread scientific and clinical adaptation of the technique, the clinical utility has to be demonstrated on a broader scale, which -given the limited availability of 7 T MRI scanners- necessitates the technique’s implementation on more widely available 3 T MRI scanners.

Tibiotalar joint injuries are common [15]. Osteochondral lesions of the talus (OLT), defined as an injury of the cartilage layer and the underlying subchondral bone, are frequent injuries in active populations that can be seen in up to 73% of all traumatic ankle injuries [16]. OLTs may predispose the joint to premature OA and ought to be diagnosed in an early and reliable manner as a timely diagnosis is a pre-requisite for appropriate treatment [17].

The aim of this study was (a) to develop and optimize a gagCEST imaging protocol for the articular cartilage of the tibiotalar joint that is clinically feasible and fits into diagnostic workflows and (b) to apply this imaging protocol to a population of healthy volunteers and patients with OLT after an ankle injury to prove clinical utility and validity. We hypothesized that -based on the developed and optimized gagCEST imaging protocol- (a) imaging of the articular cartilage of the tibiotalar joint would be possible in a clinical population and in clinically feasible time frames and (b) patients after variable ankle injuries (representative of the patient population undergoing MRI diagnostics in the clinic) demonstrate lower gagCEST effects compared to healthy volunteers.

## Methods

### Simulations

In a first step, simulations using the two-pool (water and GAG (–OH and –NH) Bloch–McConnell equation [18, 19] and a customized script (implemented in MATLAB [R2018a, The MathWorks, MA, USA] and to be downloaded at https://github.com/cest-sources/BM_sim_fit/) [20] were applied for the optimization of a pulsed gagCEST sequence [20–22]. The equations were solved analytically [19]. Based on this script, the CEST effect was simulated without the application of a saturation pulse. The radiofrequency field strength B1, the pulse duration t_p_ and the number of CEST saturation pulses n_p_ were varied using a constant duty cycle (DC) of 0.5. To keep the specific absorption rate (SAR) within the safe range, local SAR was restricted accordingly. Therefore, the maximum pulse duration was secondarily restricted by ther scanner to a maximum of 300 ms. For water, simulations were performed with relaxation times as reported earlier, i.e., T1 = 1.2 s and T2 = 0.039 s and a concentration of 88 M [23, 24]. The following parameters were used for GAG-OH protons: exchange rate = 1000 Hz, concentration 0.3 M, T1 = 1 s, T2 = 0.01 s and chemical shift = 1 ppm, and for GAG-NH protons: exchange rate = 50 Hz, concentration = 0.1 M, T1 = 1 s, T2 = 0.01 s and chemical shift = 3.2 ppm [24, 25]. The different variations of the parameters used in the simulation are displayed in Table 1; output parameters were z-spectra and MTR_asym_ curves. For each parameter, the maximum MTR_asym_ value was analytically determined at a step size of 0.01, 0.02 and 0.05 ppm at frequency offsets of 0.9–1.9 ppm, 0.5–1.5 ppm and 1–1.5 ppm. The optimized protocol in terms of the largest gagCEST effect at a reasonable acquisition time was used for the subsequent in-vivo studies.

**Table 1:** Details of sequence parameters used for simulating each parameter’s contribution to quantitatively assess GAG exchange processes based on Bloch–McConnell simulations

Experiment	*n*_p_	*t*_p_ (ms)	*B*_1_ [µT]
1	6	100	0.2; 0.4; 0.6; 0.8; 1.0; 1.2; 1.4
2	6	100; 200; 300	1.0
3	2; 4; 6; 8; 10; 12; 14	100	1.0

In each experiment, one of the three parameters (number of pulses *n*_p_, pulse duration *t*_p_, and radiofrequency-field strength *B*_1_) was systematically varied

### In-vivo study

#### Study population

19 healthy volunteers (mean age 23.0 ± 3.8, range 20–37 years, 11 males, 8 females) and six patients (mean age 31.7 ± 9.3, range 20–44 years, two males, four females) after earlier ankle injury were recruited from 06/2018 to 01/2019 via dedicated specialist consultations at our Department of Orthopedic and Trauma Surgery. The predefined inclusion criterion for patients was an isolated traumatic OLT lesion as diagnosed in earlier MRI studies. At the time of recruitment, patients were graded according to the Anderson modification of the Berndt and Harty classification and four patients had grade 1 and two patients grade 2b OLT lesions [15, 16]. Predefined exclusion criteria for healthy volunteers included all forms of primary or secondary OA of the ankle as well as other bone and joint disorders such as OLT, rheumatoid arthritis, avascular necrosis, gouty arthritis, septic arthritis, Paget disease or osteochondritis dissecans. Volunteers were also excluded if they had acute or chronic ankle pain or a history of serious trauma or surgery to the index ankle joint.

The MRI data sets of one patient and two healthy volunteers had to be excluded from image analysis due to excessive motion artifacts. The mean disease duration of patients was 22 ± 30 months (range 1–60 months). Written and informed consent was obtained from all patients before the initiation of the study. The study was approved by the local ethics committee (Ethical Committee of the University Hospital Düsseldorf, study number: 3980).

#### MRI studies

All imaging studies were performed on a 3 T MRI scanner (Magnetom Prisma, Siemens Healthineers, Erlangen, Germany) using a dedicated receive-only 16-channel foot–ankle coil (Foot/Ankle 16, Siemens Healthineers). Patients and volunteers were scanned in the supine position with a neutral ankle position of 90° dorsiflexion. Positioning aids, sandbags and medical tape were used to reduce motion artifacts.

The MRI protocol included standard morphological sequences, i.e., sagittal (sag) and coronal (cor) Proton Density-weighted (PDw) fat-saturated (fs) sequences, transversal (tra) T2-weighted turbo-spin echo (TSE), and cor T1-weighted TSE sequences. In addition to the actual gagCEST sequence as detailed below, water saturation shift referencing (WASSR), T1 mapping gradient echo (GE) and T2 multi-spin- echo (SE) mapping sequences with five different echo times (13.8, 27.6, 41.4, 55.2 and 69 ms) were acquired. Of note, the latter two sequences were only acquired in the healthy volunteers and not in the patients. GagCEST imaging was performed using a two-dimensional (2D) radiofrequency (RF)-spoiled GE sequence with a pulsed CEST pre-saturation module consisting of 8 Gaussian-shaped RF pulses with a duty cycle of 0.5. 26 images with pre-saturation pulses at different offset frequencies around the bulk water resonance were obtained. Among these images was one reference image with a frequency offset of 300 ppmThe maximum frequency offset (Δω) was 4 ppm with a step size of 0.33 ppm. In a fraction of the healthy volunteer cohort (*n* = 10, mean age 22.4 ± 1.8, range 20–25 years, seven males, three females) radiofrequency field strengths and pulse durations were systematically varied to optimize the protocol at the beginning of the study. More specifically, three different radiofrequency field strengths (*B*_1_ = 0.6, 0.8 and 1.0 µT) and three different pulse durations (*t*_p_ = 100, 200 and 300 ms) were used. Based on the results of the simulations, i.e., the largest measured MTR_asym_ values, we used a radiofrequency field strength of *B*_1_ = 0.8 and a pulse duration *t*_p_ = 300 ms in the remaining healthy volunteer and patient cohorts. For the WASSR sequence, 22 images with pre-saturation and a reduced radiofrequency field strength (*B*_1_ = 0.25 µT) were obtained. The maximum frequency offset was decreased to Δω = 1 ppm with a step size of 0.1 ppm. For WASSR and CEST sequences, motion correction was applied. The acquisition time was 5:01 min for the CEST and 2:22 min for the WASSR sequence. The total acquisition times for the compositional MRI sequences were: 24:21 min for the initial 10 healthy volunteers (3 × 5:05 min CEST, 1 × 2:22 min WASSR, 6 × 1:14 min T1) and 7:27 min for the remaining 7 healthy volunteers and the 5 patients (1 × 5:05 min CEST and 1 × 2:22 min WASSR). The acquisition time for the morphological sequences was 18 min, resulting in a total scan time of 42:21 min for the initial 10 volunteers and 25:27 min for the consecutive 7 volunteers and the 5 patients.

Detailed parameters of the morphological and compositional sequences are given in Tables 2 and 3.

**Table 2:** Detailed sequence parameters of morphological MRI sequences

Imaging parameter	Sagittal fs PDw	Coronal fs PDw	Transversal T2w TSE	Coronal T1w TSE
FOV (mm)	160 × 160	160 × 160	160 × 160	160 × 160
Slice thickness (mm)	3	3	3	3
TE (ms)	40	40	78	17
TR (ms)	4000	4000	4600	700
Resolution (mm/pixel)	0.31 × 0.42	0.31 × 0.42	0.31 × 0.39	0.28 × 0.4
Flip angle (°)	150	150	150	140
Acquisition matrix	512 × 384	512 × 384	512 × 410	576 × 403

Field of view (FOV), slice thickness, echo time (TE), repetition time (TR), resolution, flip angle, and acquisition matrix are given for sagittal and coronal fat-saturated proton-density-weighted (fs PDw), transversal T2-weighted turbo spin echo (T2w TSE) and coronal T1-weighted TSE (T1w TSE) sequences

**Table 3:** Detailed sequence parameters of compositional MRI sequences

Imaging parameter	WASSR	gagCEST	T1 map	T2 map
FOV (mm)	160 × 160	160 × 160	160 × 160	160 × 160
Slice thickness (mm)	5	5	7	3 mm
TE (ms)	3.5	3.5	11	13.8/27.6, 41.4/55.2/69
TR (ms)	7.2	7.2	6000	1000
TI (ms)			25/50/100/500/1000/2000	
Resolution (mm/pixel)	0.6 × 0.6	0.6 × 0.6	0.6 × 0.6	0.4 × 0.4
Flip angle (°)	15	15	180	180
Pulsed CEST saturation module				
Frequency range (ppm – ppm)	− 1 to 1	− 3 to 3		
Number of Dynamic Scans	21 + 1	25 + 1 reference image		
Number of saturation pulses	1	8		
Pulse Duration *t*_p_ (ms)	54	300 (100, 200)		
Interpulse Duration (ms)	–	300		
*B*_1_ amplitude (µT)	0.2	0.8 (0.6, 1.0)		

In healthy volunteers, pulse duration *t*_p_ and *B*_1_ amplitude were evaluated at 100, 200, and 300 ms and at 0.6, 0.8, and 1.0 µT, respectively, while in patients, the following parameter settings were used: 300 ms and 0.8 µT

*WASSR* water saturation, *gagCEST* glycosaminoglycan chemical exchange saturation transfer imaging, *FOV* field of view, *TE* echo time, *TR* repetition time, *TI* inversion time

#### Image analysis

**Fig. 1:**
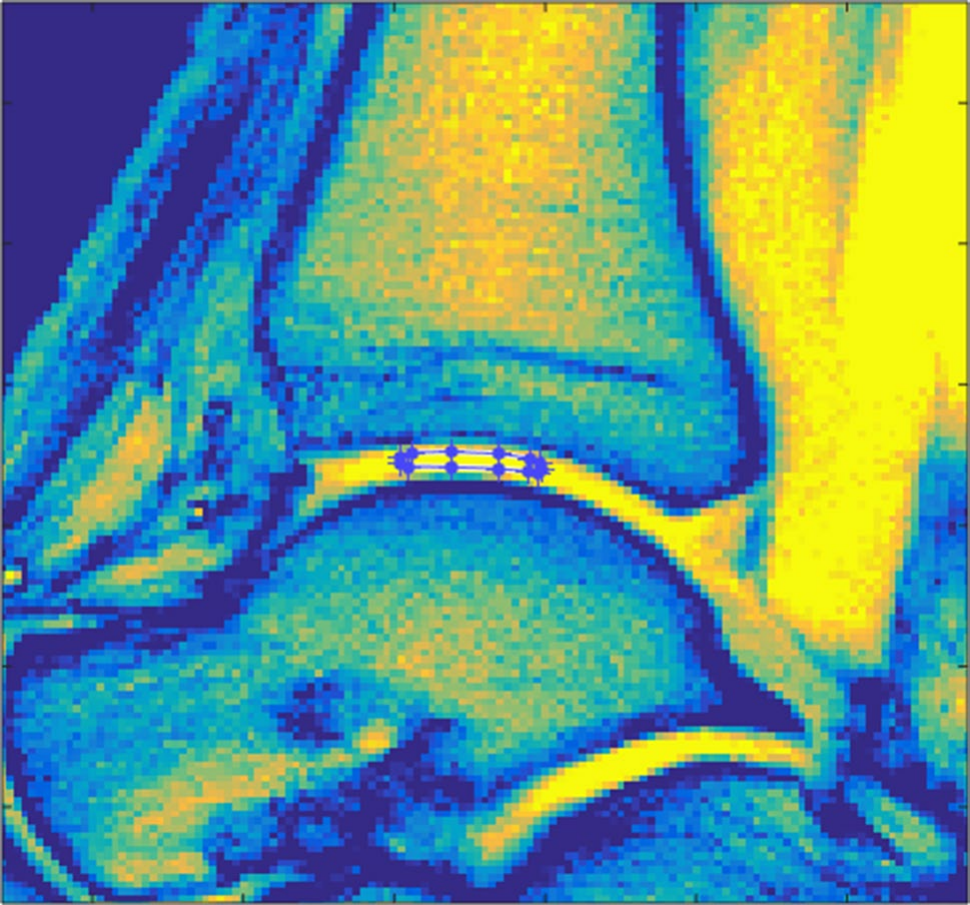
Exemplary image detailing the region-of-interest (ROI) definition. Water saturation shift referencing sequence (WASSR) image of the tibiotalar joint of a 29-year-old healthy male. Manual definition of the ROI in the central weight-bearing region of the tibiotalar joint was performed individually by two radiologists to include the cartilage layers of the tibiotalar joints while reducing partial volume artifacts due to cortical bone and/or joint fluid

All images were independently analyzed by two radiologists (DBA, 3 years of training in musculoskeletal imaging; CS, 8 years of training in musculoskeletal imaging) who were blinded to the volunteers’ or patients’ data. First, all studies were read to determine the individual joint’s overall status with a particular focus on the integrity of tibiotalar cartilage. Also, OLTs were -if present- classified according to Hepple et al. [26]. Second, using the unsaturated WASSR image, both readers independently identified the cartilage layers of the tibiotalar joint and quantified its biophysical properties in a standardized manner by placing an ellipsoid-shaped region-of-interest (ROI) in the median plane onto both cartilage layers at the central load-bearing region of the tibiotalar joint. Each ROI was placed distant to the tibial and talar bone cortex and the anterior and posterior joint areas to reduce partial volume artifacts due to the presence of cortical bone and potentially excessive amounts of joint fluid (Fig. 1). The second reader repeated the ROI placement at a different time point to allow for the assessment of inter-rater reliability.

For the analysis of the MTR_asym_ curve, i.e., the CEST effect, we used an in-house script implemented in Matlab (MATLAB R2018a, The MathWorks, Inc., MA, USA). Prior to further evaluation, B_0_ field inhomogeneities were corrected by the WASSR maximum-symmetry algorithm with the calculation of a pixel-wise frequency offset curve [27, 28]. These offset-corrected CEST-curves divided by the signal without pre-saturation (*S*_0_) were defined as the so-called z-spectrum (*Z* (ω)). The maximum frequency offset of each z-spectrum was Δω = 3 ppm. Next, we used the magnetization transfer asymmetry (MTR_asym_) (defined as MTR_asym_(Δω) = Z(− Δω) − Z(Δω)) for the evaluation of the gagCEST effect [29]. MTR_asym_ maps were calculated using the average value of MTR_asym_ in the GAG-specific range of Δω = 0.9 − 1.9 ppm [30]. In addition, the *B*_0-_ corrected and -normalized spectra were fitted using Lorentzian function analysis to account for the GAG-OH, GAG-NH, water pools at − 1 ppm, the nuclear Overhauser effect at − 1 and − 2.8 ppm and the magnetization transfer pool at − 2.43 ppm [31, 32]. In the following, the Lorentzian-fitted gagCEST effect is given as GAG-OH amplitude.

T1 and T2 relaxation times calculations in ten healthy volunteers were also performed in Matlab. In a pixel-wise manner, acquired data was fitted and calculated based on the following equations:

$$T1{: M}_{z}\left(t\right)={M}_{z}^{0}-\left({M}_{z}^{0}-{M}_{z}\left(0\right)\right)exp(\frac{-t}{{T}_{1}})$$

$$T2{: M}_{xy}\left(t\right)={M}_{xy}\left(0\right)exp(\frac{-t}{{T}_{2}})$$

with T1 and T2 being the sought relaxation times, ${M}_{z}\left(t\right)$ the total magnetization in the *z*-direction, and ${M}_{xy}\left(t\right)$ the total magnetization in the *xy*-plane at time point *t*.

#### Statistical analysis

SPSS software (IBM, version 22, Armonk, NY, USA) was used for all statistical analyses by KLR and DBA. For descriptive analysis, mean gagCEST values ± standard deviation, median, and range (minimum–maximum) were calculated for healthy volunteers and patients. For optimization of the imaging protocol radiofrequency field strength and pulse duration were systematically varied and then compared using a multivariate analysis of variance (MANOVA) and a post-hoc Scheffé-test. For the comparison of gagCEST values between both cohorts, a multivariable statistical analysis was performed using a linear mixed model (LMM). The established model included a subject-specific random intercept, the factors healthy volunteer/patient, age, gender and the interaction of these factors assuming a fixed linear effect on the gagCEST values. Results of this model are given in Table 1 of the Supplementary Material. The LMM was fitted using a restricted maximum likelihood approach (REML). Based on this final model, the mean differences of gagCEST values were calculated and evaluated for significance. For correlation analyses of MTRasym values and GAG-OHamplitudes, Pearson’s correlation was determined and quantified using the correlation coefficient r. Correlation strength was graded as suggested by Cohen [33]: small (0.1–0.3), moderate (0.3–0.5), and large (> 0.5). *p* values < 0.05 were considered significant. For the evaluation of inter- and intrarater reliability, single and average measure intraclass correlation coefficients (sICC and aICC) were calculated based on the ROIs drawn by the two raters.

## Results

### Simulations

**Fig. 2:**
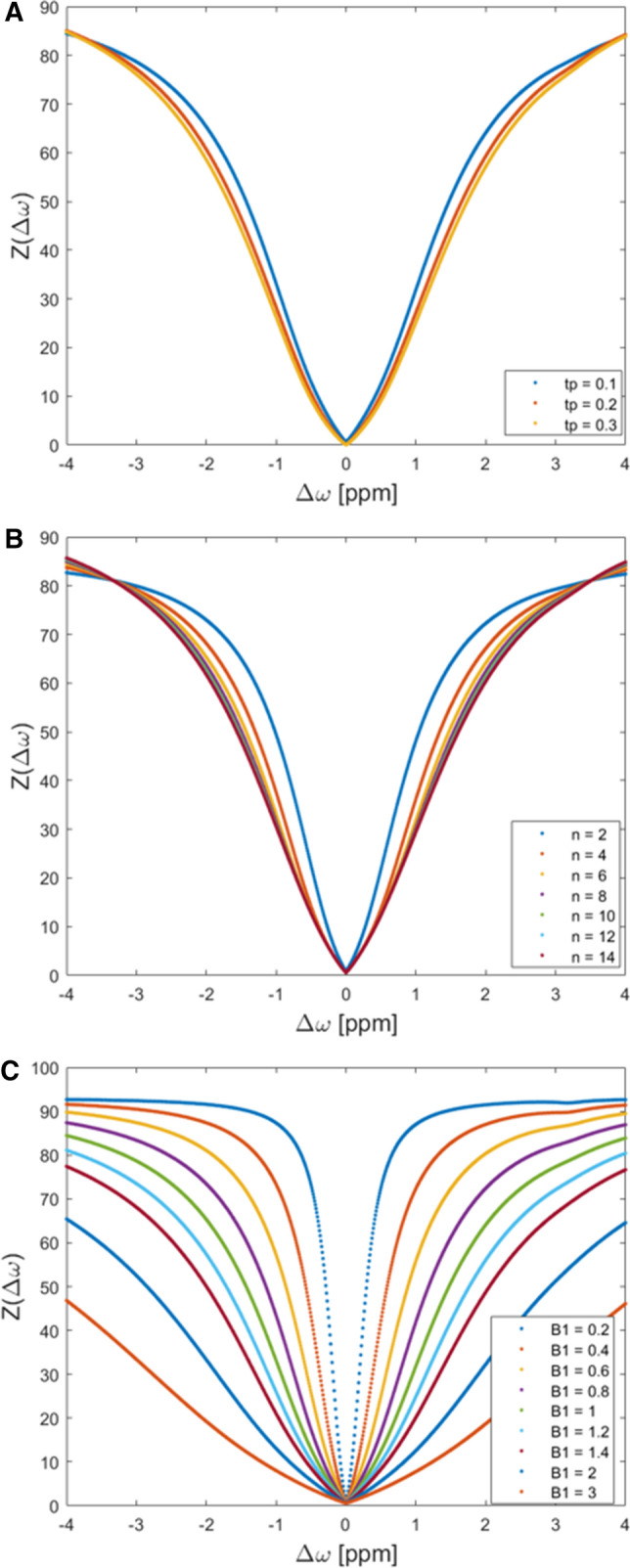
Simulations results detailing the effects of variations in CEST framework conditions. Pulse durations *t*_p_ (100, 200, and 300 ms) (**a**), number of applied pulses n_p_ (*n* = 2 – 14) (**b**), and radiofrequency field strengths *B*_1_ (0.2–3 µT) (**c**) were systematically varied. Each colored curve represents a simulated parameter value and gives the z-spectrum at different offset frequencies (0–4 ppm)

**Fig. 3:**
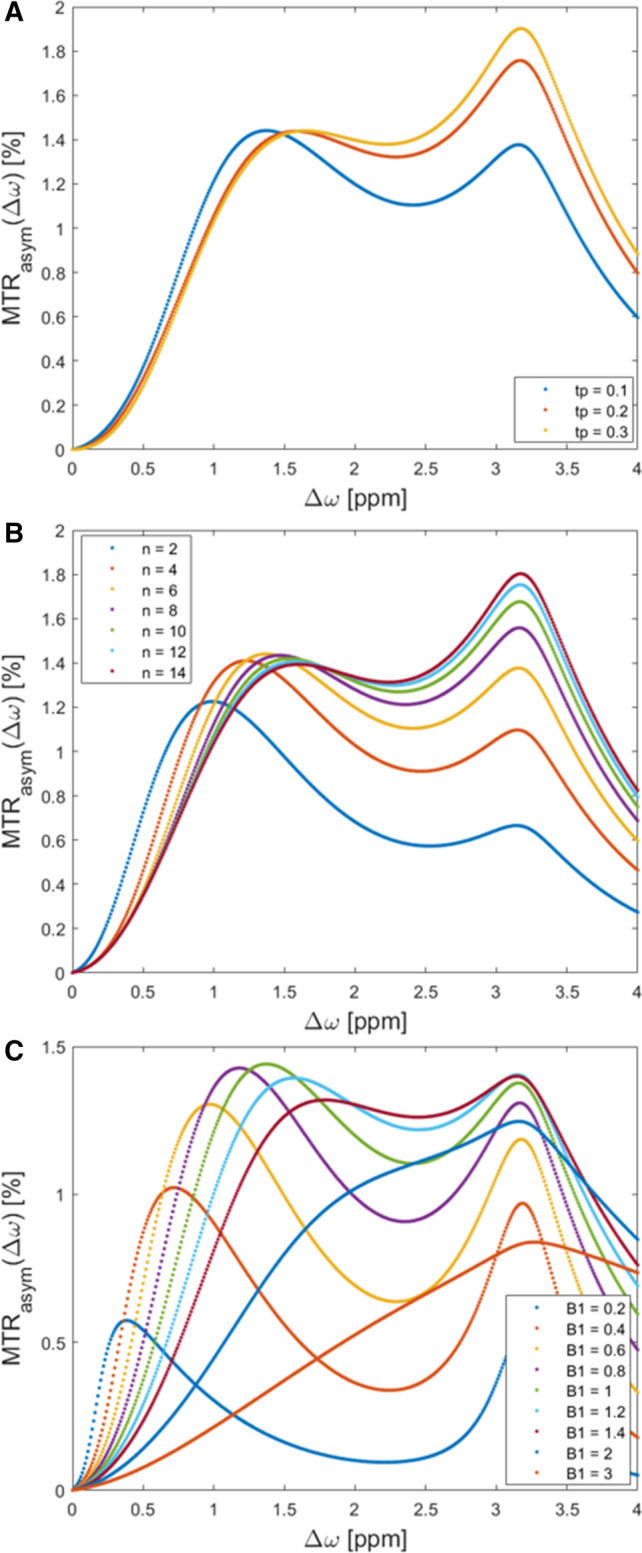
Simulations results detailing the effects of variations in CEST framework conditions. Pulse durations *t*_p_ (100, 200, and 300 ms) (**a**), number of applied pulses n_p_ (*n* = 2 – 14) (**b**), and radiofrequency field strengths *B*_1_ (0.2–1.4 µT) (**c**) were systematically varied. Each colored curve represents a simulated parameter value and gives the maximum magnetization transfer ratio asymmetry at different offset frequencies (0–4 ppm)

The results of the systematic simulations are illustrated in Figs. 2 and 3.

- Variation of *t*_p_.
  Maximum MTRasym values were 1.33 % at 0.9–1.9 ppm with *t*_p_ = 200 ms, 1.07 % at 0.5–1.5 ppm with *t*_p_ = 100 ms and 1.37 % at 1.0–1.5 ppm with *t*_p_ = 100 ms (Fig. 3a).
- Variation of *n*_p_.
  The CEST effect increases with the number of applied saturation pulses (*n*_p_*)* (Fig. 3b). Eight applied pulses reach 98% of the maximum gagCEST effect that could be obtained with 14 pulses at all ranges (0.9–1.9 ppm, 0.5–1.5 ppm and 1.0–1.5 ppm). Maximum MTR_asym_ values with eight applied pulses were 1.33 % at 0.9–1.9 ppm, 1.02 % at 0.5–1.5 ppm and 1.33 % at 1.0–1.5 ppm.
- Variation of *B*_1_.

The CEST effect increases with increasing *B*_1_ until it reaches a maximum (Fig. 3c). Due to the spillover effect, MTR_asym_ values decrease beyond the maximum. Maximum MTR_asym_ values were 1.33 % at 0.9–1.9 ppm and a *B*_1_ of 1 µT, 1.17 % at 0.5–1.5 ppm and a *B*_1_ of 0.8 µT and 1.37 % at 1.0–1.5 ppm and a *B*_1_ of 1 µT.

### In-vivo studies

#### Morphological MRI of patients and healthy volunteers

Apart from the presence of OLTs as outlined below and a moderate joint effusion, the overall joint status of three of five patients was unremarkable. In them, we did not find any bone marrow lesions, subchondral thickening, osteophytes or joint space narrowing. In two patients, we noted signs of secondary OA with osteophytes, joint space narrowing, subchondral sclerosis, and moderate joint effusion. The joint status of healthy volunteers was unremarkable without any structural alterations. Within the entire study population, the following accessory ossicles were found: Os tibiale externum in six individuals, Os trigonum in three individuals, Os supratalare in one individual.

Staging of OLTs was performed according to the Heppner classification (stages 1–5, 1: articular cartilage damage only, 2a: cartilage injury with underlying fracture and surrounding edema, 2b: 2a without surrounding edema, 3: detached, but undisplaced fragment, 4: detached and displaced fragment, 5: subchondral cyst). The following stages were observed in the patient cohort: one individual with stage 2a, one individual with stage 3, one individual with stage 4 and two individuals with stage 5.

#### Implementation of the optimized protocol in 10 healthy volunteers

Table 4 gives the details of the MTRasym values in 10 healthy volunteers as a function of systematically varied parameter settings of *B*_1_ (0.6, 0.8, and 1.0 µT) and tp (100, 200, and 300 ms).

- Variation of *B*_1_.
  The mean MTR_asym_ values had a maximum of 1.7 ± 1.4 % at 0.8 µT and tended to be -even though non-significantly- numerically higher than at 1.0 µT (0.5 ± 1.0 %, *p* = 0.073) and at 0.6 µT (1.3 ± 1.1 %, *p* = 0.759).
- Variation of *t*_p_.

**Table 4:** Magnetization transfer ratio asymmetry (MTR_asym_) values as a function of systematically varied *B*_1_ and *t*_p_ in 10 healthy volunteers

Offset frequency [ppm]	*B*_1_ (µT)	*t*_p_ (ms)	MTRasym (%)	*p* value
0.9–1.9	0.6	100 200 300	0.37 ± 0.78 0.75 ± 0.65 1.34 ± 1.05	100 vs. 200 ms: < **0.001** 100 vs. 300 ms: < **0.001** 200 vs. 300 ms: **0.016** 0.6 vs. 0.8: 1.0 0.6 vs. 1.0: **0.001** 0.8 vs. 1.0: < **0.001**
	0.8	100 200 300	0.12 ± 0.47 0.71 ± 0.81 1.67 ± 1.35	
	1.0	100 200 300	0.27 ± 0.78 0.94 ± 1.02 0.49 ± 0.95	

MTR_asym_ values are given as mean ± standard deviation

Means were compared using a multivariate analysis of variance (MANOVA) followed by a post-hoc Scheffé test

*p* values < 0.05 were considered significant and are given in bold type

The highest mean MTR_asym_ values were found at *t*_p_ = 300 ms that were significantly higher than at *t*_p_ = 100 ms (1.67 vs. 0.12 %, *p* < 0.004) and tended to be higher than at *t*_p_ = 200 ms (1.67 vs 0.71 %, *p* = 0.092).

#### Implementation of the optimized protocol in all healthy volunteers and patients

**Fig. 4:**
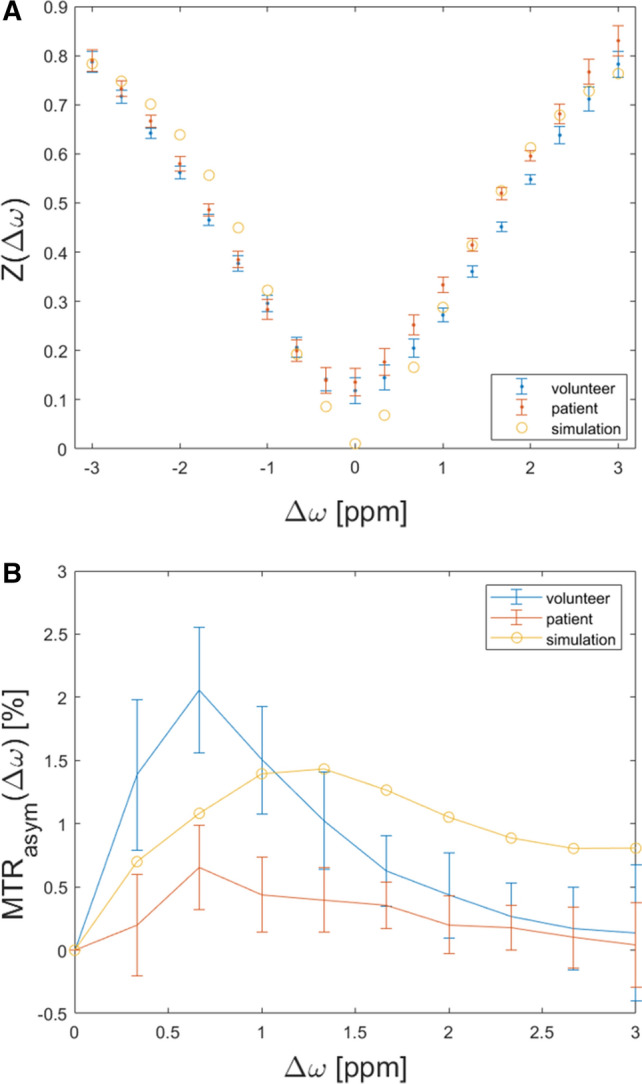
Illustration of Z-spectra (**a**) and MTRasym (**b**) curves of the simulation (blue), a volunteer (yellow) and a patient (orange)**.** CEST framework conditions were *B*_1_ = 0.8, *t*_p_ = 300 ms and np = 8. Simulations results For the patient’s and volunteer’s curves means (dots) and standard deviations (whiskers) are given.Of note, the GAG-NH peak is only visible in the simulation, but not in-vivo
  Gag-OH amplitudes of the tibiotalar joint cartilage in patients were 0.8±0.4 % (95% CI 0–1.6) and in healthy volunteers 2.0±0.2 % (CI 1.6–2.4) (*p* = 0.013). We found strong and significant correlations between mean MTR_asym_ values and gagOH amplitudes (*r*= 0.56, *p* = 0.006).
  No significant differences were found between the volunteers that were used for protocol optimization and the remaining volunteers (volunteer cohort 1: MTRasym: 1.5 ± 0.9 %, volunteer cohort 2: MTRasym: 1.4 ± 0.9 %, *p* = 0.715).
  The reproducibility of the MTR_asym_ values of all ROIs was excellent (aICC= 0.97, 95% confidence intervals 0.82/0.95, *p* < 0.001 and sICC= 0.91, 95% CI 0.93/0.98, *p* < 0.001).
- T1 and T2 relaxation times in healthy volunteers.

- MTR_asym_ values and GAG-OH amplitude of healthy volunteers vs. patients.
  Using the optimized imaging protocol (with the following framework conditions: radiofrequency-field strength *B*_1_= 0.8, pulse duration *t*_p_= 300 ms and number of pulses *n*_*p*_= 8), the mean MTR_asym_ value of the tibiotalar joint cartilage in patients was 0.3 ± 0.2 % (95 % confidence interval [CI] 0–0.7) and in healthy volunteers was 1.5 ± 0.9 % (95 % CI 1.3–1.7) (*p* < 0.001). MTR_asym_ values are visualized in Fig. 3. Corresponding gagCEST maps are given in Fig. 4.

**Fig. 5:**
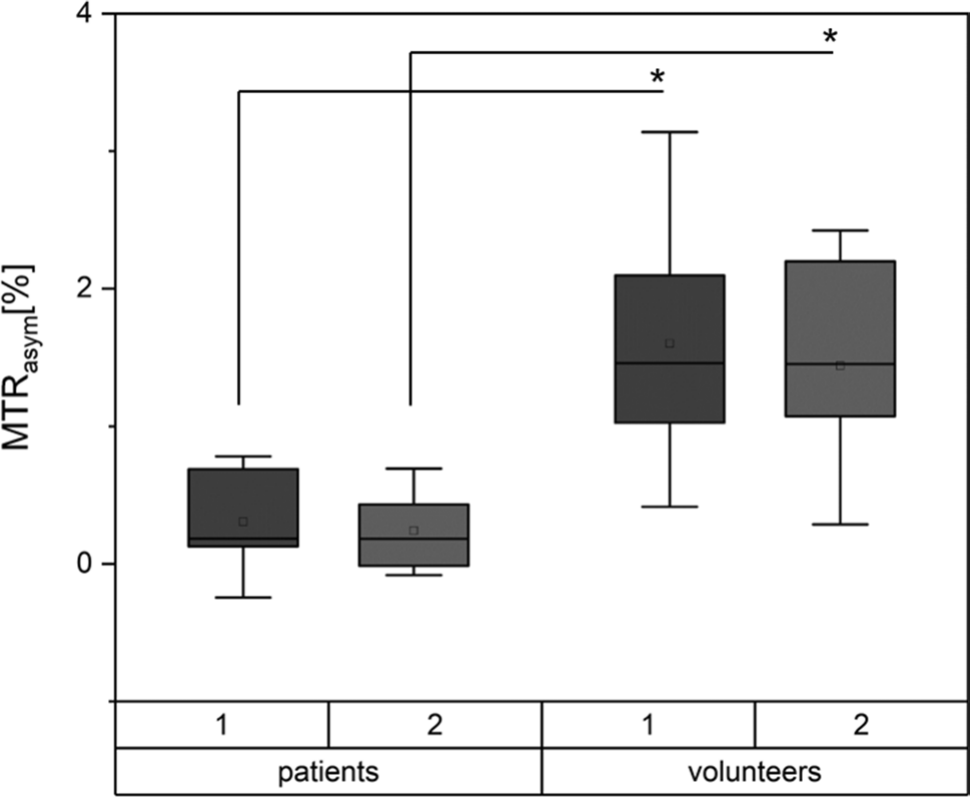
Comparison of MTR_asym_ values in patients and healthy volunteers. Data are presented as means (thick line), medians (square boxes), standard deviation (boxes), and ranges (whiskers). For each cohort, two separate boxes are presented: 1 gives the MTRasym values of the ROI defined by rater 1. Box 2 depict the MTR_asym_ values of the corresponding ROIs of rater 2. *p* values < 0.05 were considered significant and are highlighted with an asterisk

**Fig. 6:**
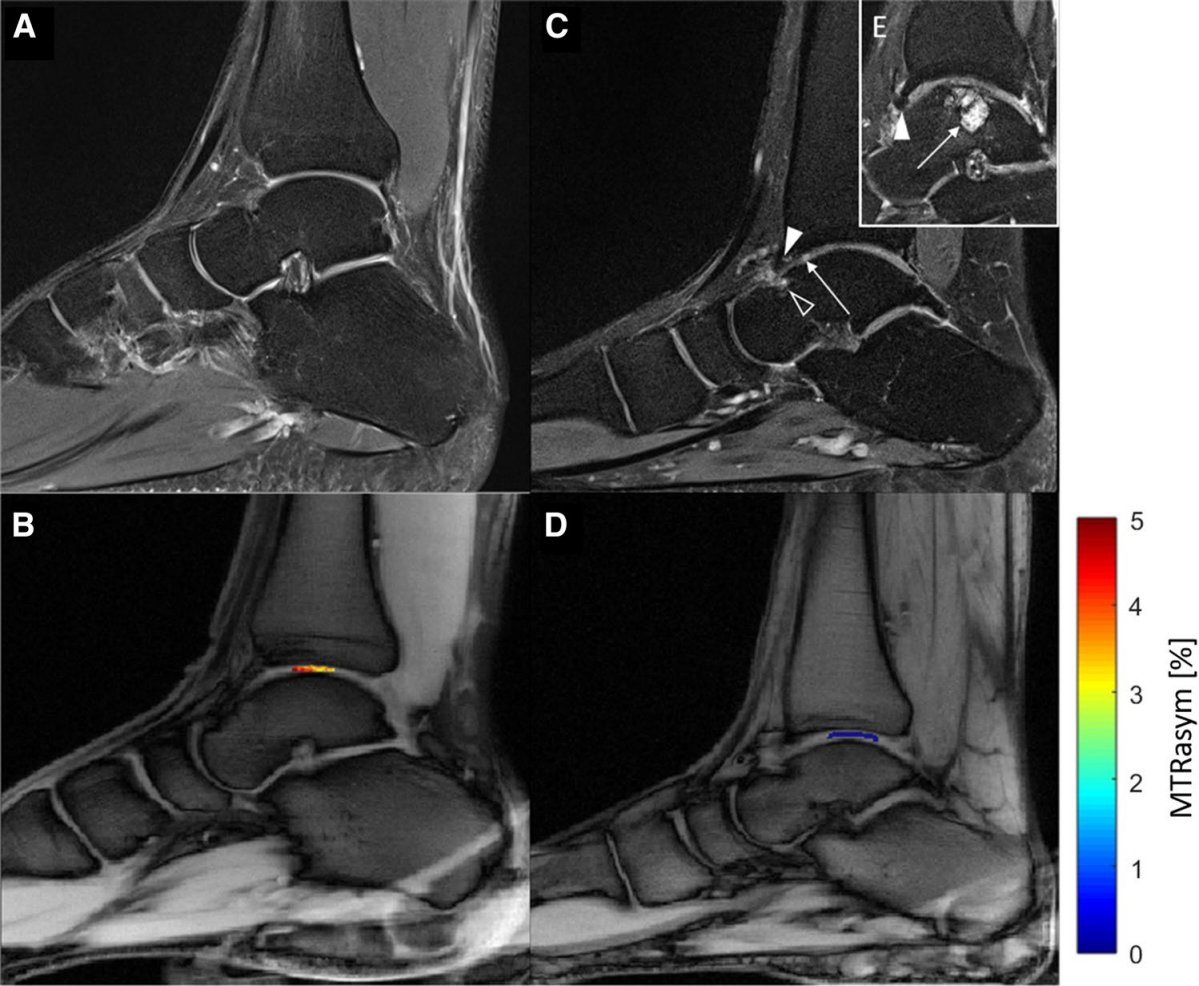
Sagittal proton-density weighted (PDw) images and corresponding glycosaminoglycan chemical exchange saturation transfer (gagCEST) maps of a 29-year-old healthy male (**a** and **b**) and an age-matched male patient with an established osteochondral lesion of the talus (OLT; **c**, **d**, **e**). **a** Unremarkable tibiotalar joint with no sign of cartilage damage, osteoarthritis or OLT. **c** Osteochondral lesion of the anterior talus (black arrowhead), osteophyte of the anterior tibia (white arrowhead), and intra-tissue signal hyperintensity of the anterior tibiotalar joint cartilage indicative of focal cartilage damage (long arrow). **e** More medial to (**c**), presence of a large cystic OLT in the weight-bearing aspect of the talus (long arrow) representing a stage 5 OLT according to the Hepple classification and an osteophyte of the anterior tibia (arrowhead). Overall, the tibiotalar joint cartilage is focally thinned, inhomogeneous, and irregular. **b** and **d** The tibiotalar joint cartilage of the healthy volunteer has higher gagCEST values than the patient (color-coded gagCEST maps overlaid onto T1w morphological image)

The in-vivo measurements in healthy volunteers showed a mean T1 relaxation time of 940 ± 120 ms (range 720–1080 ms) and a mean T2 relaxation time of 35± 7 ms (range 26–48 ms) (Figs. 5, 6).

## Discussion

The most important finding of this study is that -following comprehensive and systematic sequence optimization- gagCEST imaging of the tibiotalar joint is feasible using a clinical standard 3 T MRI scanner, fits into clinical workflows with an acquisition time of less than 07:30 min, and yields stable and reproducible results that allow compositional cartilage assessment. In addition, we demonstrated that the tibiotalar joint cartilage of patients with known tibiotalar joint injury, especially OLT, have significantly lower gagCEST values than healthy volunteers.

Compositional MRI exceeds the mere morphological depiction of cartilage and allows for the detection of early cartilage changes that precede morphological alterations, i.e., loss of proteoglycans, as an early, potentially treatable stage of OA. GagCEST can be used for the detection and treatment monitoring of very early OA [34]. Despite this great clinical need, research on gagCEST imaging in general has been limited by the numerous technical complexities involved such as homogeneous magnetic field properties, long scan times, low SNR, and high field strengths (optimally ≥ 7.0 T) that are considered necessary for cartilage imaging [35]. Moreover, with the majority of imaging studies focusing on the knee joint, data on the tibiotalar joint is sparse [14]. This is mainly due to the joint’s limited cartilage thickness, measuring only about 2 mm in healthy individuals and the known limited spatial resolution of gagCEST imaging [36, 37]. In this study, we set out to establish and optimize a gagCEST imaging protocol with reasonable scan times, sufficient SNR, and high reproducibility at 3.0 T for the potential implementation in the clinical setting.

GagCEST imaging can be modified by altering the number of applied saturation pulses, pulse durations and radiofrequency field strengths. To find the optimal setting of these parameters that allow for both a high gagCEST effect size and reasonable acquisition time, we used the Bloch–McConnell simulation before proceeding with the *in-vivo* measurements [38]. The simulation experiments showed a maximum effect size at a radiofrequency field strength of 0.8 µT. The effect size decreased at higher field strengths due to the *‘spillover effect’*: With an increasing *B*_1_ amplitude, the spillover effect leads to direct saturation of the water pool instead of the soluble proton pool and hence results in decreases of the gagCEST effect [39]. When tested in healthy volunteers, we noted numerically higher MTR_asym_ values and GAG-OH amplitudes at 0.8 than at 1.0 µT, but not than at 0.6 µT. The effect size increased with the applied number of pulses with a MTR_asym_ of 0.98% at 14 pulses; however, at eight applied pulses, the MTR_asym_ reached 0.98% of the maximum effect size. To keep the acquisition time as short as possible at a maximum gagCEST effect size, we decided to use eight pulses. Moreover, the effect size was found to be increased with increasing pulse durations. Due to limitations secondary to the specific absorption rate (SAR); however, the maximum pulse durations to be used in vivo were limited to 300 ms [40]. By trend, we found higher MTR_asym_ values in vivo at a pulse duration of 300 ms (as compared to 100 and 200 ms)—even though these differences were only partially significant.

After simulations and in-vivo experiments, our final gagCEST protocol consisted of 8 applied pulses with a pulse duration of 300 ms at a radiofrequency field strength of 0.8 µT and a constant duty cycle of 0.5 aiming for a minimized scan time. We used WASSR to improve the differentiation of the water and GAG peak as well as to correct for B_0_ field inhomogeneities [27]. Using this protocol, we found excellent reproducibility of gagCEST values as measured by one individual rater and between two independent raters (aICC = 0.97 and sICC = 0.91). These values for reproducibility were even higher than presented in previous studies focusing on gagCEST of peripheral joints [34]. A good reproducibility is beneficial not only for future studies, but also for the perspective of clinical implementation of the technique [41].

The acquisition time of the optimized gagCEST sequence was 5:01 min, followed by an additional 2:22 min for the WASSR sequence. Thus, the sequence requires 7:23 min. Hence, our scan time is comparable to the one presented by Kogan et al., who conducted the only previous study on gagCEST imaging of the ankle joint, and even shorter than several gagCEST studies focusing on the knee joint [13, 14, 42]. Additionally, the gagCEST imaging protocol was designed for 3 T scanners, which is the commonly used field strength for musculoskeletal imaging in clinical practice [43]. Thus, our protocol may be applied in both research and clinical contexts to further advance the clinical utility of gagCEST imaging of the tibiotalar joint. However, it still has to be considered less sensitive at detecting early cartilage changes than imaging protocols applied at 7 T scanners, especially if the latter are designed as volumetric multi-slice approaches [14]. Volumetric protocols have been implemented at 3 T scanners for gagCEST imaging of the knee joint and generally allow for better localization of cartilage changes. Consequently, future adaptation of volumetric protocols for gagCEST imaging of the tibiotalar joint seems of great scientific and clinical interest.

In addition to providing a stable and reproducible protocol, we observed significant differences between healthy volunteers and patients with OLTs. Since this study was the first of its kind comparing healthy individuals with patients using gagCEST at the ankle joint, we chose a patient cohort with morphologically damaged cartilage to demonstrate feasibility of this technique. In the future, we intend to study patients after ankle trauma without morphological apparent cartilage lesions to assess the presence of pre-morphological tissue damage.

Despite its strengths, our study has limitations. Our measured T1 and T2 relaxation times were shorter than the ones used for the simulations, but were overall comparable to the current literature [44].

Synovial fluid in general and joint effusion in particular are known to interfere with gagCEST imaging due to the presence of GAGs [1, 45]. Therefore, we placed our ROIs in the center of the tibiotalar joint at a distance to the anterior and posterior anatomical recesses, where joint fluid may collect and distort our measurements. Á priori, we excluded patients with manifest joint effusion as visible in the morphological sequences. However, since we included both cartilage layers, i.e., both tibial and talar, in one single ROI, the odds are high that synovial fluid might have contaminated our gagCEST measures. Future studies should, therefore, use sequences that use fluid suppression. Moreover, our study population was small, which may be explained by the fact that we set out to implement a clinically applicable imaging protocol for gagCEST imaging. Nonetheless, future studies need to be conducted to corroborate our findings in larger patient numbers. Furthermore, we did not compare our findings to the gold-standard technique dGEMRIC. Since dGEMRIC relies on gadolinium-based contrast agents and its use is restricted due to ethical reasons, we consider this only a minor limitation. Last, we used a two-pool exchange model considering only the water- and the GAG-OH pool for the simulation. This model might be partially inaccurate for *in-vivo* applications, because of other influencing factors such as the GAG-NH pool, the nuclear Overhauser effect (NOE), and the magnetization transfer (MT) that were not included in our simulation because of lacking application-specific-framework fitting parameters for the NOE and MT. However, for the eventual quantification of the *in-vivo* measurements we used both the MTRasym values and the Lorentzian fit analyses. While the former accounts only for the water and the GAG-OH pool the latter also takes the GAG-NH, NOE and magnetization transfer pools into consideration. As both were strongly correlated, we consider the morge simple two-pool exchange model to be sufficient for *in-vivo* quantification purposes.

In this feasibility study, pre-morphological tibiotalar joint cartilage damage was quantitatively assessable on the basis of an optimized 3 T gagCEST imaging protocol that allowed a stable gagCEST effect quantification both in normal and degenerated cartilage in clinically feasible acquisition times.

## Electronic supplementary material

Below is the link to the electronic supplementary material.

Supplementary file1 (DOCX 18 kb)
