# Supplementary material for: Detection of early cartilage degeneration in the tibiotalar joint using 3 T gagCEST imaging: a feasibility study
Source: MAGMA. 2020 Jul 28;34(2):249–60. doi: 10.1007/s10334-020-00868-y (PMC8018923; doi:10.1007/s10334-020-00868-y)
Supplement: Supplementary file 1 — Supplementary file1 (DOCX 18 kb) [file 10334_2020_868_MOESM1_ESM.docx]

|  | |  | |  | |  | |  | | 95% Confidence Interval | |
| --- | --- | --- | --- | --- | --- | --- | --- | --- | --- | --- | --- |
| Parameter | Estimate | | Standard error | | Degrees of freedom | | T-statistic | | P-value | Lower | Upper |
| Y-intercept | 1.58 | | 0.49 | | 66 | | 3.21 | | 0.002 | 0.60 | 2.57 |
| [volunteer =patient] | -1.15 | | 0.24 | | 66 | | -4.89 | | <0.001 | -1.63 | -0.68 |
| [sex=male] | 0.28 | | 0.20 | | 66 | | 1.43 | | 0.157 | -0.11 | 0.68 |
| Age | -0.01 | | 0.02 | | 66 | | -0.73 | | 0.468 | -0.05 | 0.02 |

**Table 1** Estimates of fixed parameters on MTR_asym_ values based on a linear mixed model including a subject-specific random intercept, the facots volunteer/patient, age, gender and the interaction of these factors assuming a fixed linear effect on the gagCEST values.
